# Photoinduced refractive index variation within picosecond laser pulses excitation as the indicator of oxyorthosilicates single crystals composition modification

**DOI:** 10.1186/s11671-015-0799-1

**Published:** 2015-03-01

**Authors:** Andrii V Uklein, Alexandr S Popov, Volodymyr V Multian, Mykhaylo S Brodyn, Valerii V Kononets, Oleg Ts Sidletskiy, Volodymyr Ya Gayvoronsky

**Affiliations:** Department of Nonlinear Optics, Institute of Physics NAS of Ukraine, pr. Nauki, 46, Kiev, 03680 Ukraine; Department of Crystal Growth Technology, Institute for Scintillation Materials NAS of Ukraine, pr. Lenina, 60, Kharkiv, 61001 Ukraine

**Keywords:** Scintillators, Nonlinear optical response, Oxygen vacancies, Resonant pulse excitation, Crystals composition modification diagnostics

## Abstract

For the first time, the diagnostics of oxyorthosilicates single crystals based on self-action of picosecond range laser pulses at 1,064 nm (1.17 eV) has been performed. High sensitivity of the photoinduced refractive index variation to the substitution of the Lu atoms by Gd in the LSO/LGSO crystalline host as well as to the admixture of Ce was found. The effect can be explained with different electron detrapping-recombination process efficiencies due to the resonant electron excitation from the deep traps in the gap attributed to intrinsic oxygen vacancies.

## Background

The scintillators based on oxyorthosilicate single crystals are under the interest due to their potential application in the field of nuclear medicine [1] and high energy physics [2]. Ce ^3+^ plays the role of luminescence activator in many scintillation materials. In Lu _2*x*_*Gd*_2−2*x*_*SiO*_5_:Ce (LGSO:Ce), incorporation of Gd ^3+^ ions into the host is one of the ways to improve scintillation properties of a well-known Lu _2_*SiO*_5_ (LSO) scintillator [3]. Mechanisms of light yield improvement in LGSO:Ce are still under discussion. It can be attributed to the carrier migration lengths reduction due the nano- or microsize spatial inhomogeneities formation in the crystals enriched with Gd ^3+^ or Lu ^3+^ ions. The inhomogeneities were observed experimentally on the microscale level [4]. They induce the spatial local fluctuations of the conduction and valence bandgap edges that cause a decrease of the stochastic recombination of electrons and holes, a promotion of the carriers localization within the same fluctuation, and their transport to the activator ions [5]. In other words, the introduction of the dopants leads to modification of the detrapping-recombination effects in the crystals that can be characterized by nonlinear optical (NLO) response analysis within a picosecond range pulsed laser radiation self-action technique at 1,064 nm (1.17 eV). A sensitivity of the method is based on resonant electron excitation from the deep traps in the gap attributed to intrinsic oxygen vacancies [6] in the studied materials. The technique has been approved for metal oxide nanocomposite characterization with the similar oxygen vacancy occurrence depth in the gap [7,8].

## Methods

### The crystals growth and characterization

**Figure 1:**
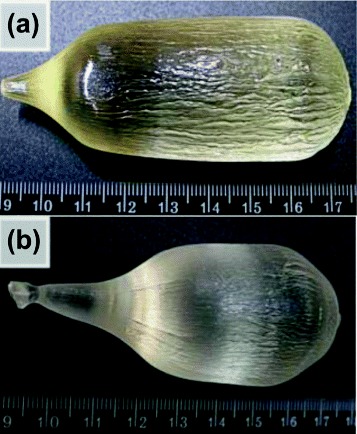
**Photos of as grown orthosilicate boules: LSO doped with Ce ions (a); LGSO doped with Ce ions (b).**

Rare-earth oxides Lu _2_*O*_3_, Gd _2_*O*_3_, CeO _2_, and SiO _2_ with purity not worse than 4 N mixed at stoichiometric ratios were used as starting materials for crystal growth. Crystals with the diameter up to 35 and the length up to 50 mm (Figure 1) were grown using the Czochralski method in Ir crucibles of 60 mm diameter and 60 mm height. Post-growth annealing of the ingots was carried out in the inert atmosphere at 1,500°C [3]. From the grown boules, the samples with approximately 1 mm thickness were cut and polished. The concentration of Ce was 0.2 at% in LSO:Ce crystal and − 0.5 at% in LGSO:Ce one.

**Figure 2:**
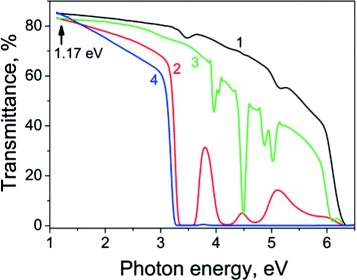
**The transmittance spectra of the orthosilicate single crystals: 1 – LSO, 2 – LSO:Ce, 3 – LGSO, 4 – LGSO:Ce.** The arrow indicates the excitation photon energy in the crystals transparency range.

The transmittance spectra of the studied crystals are presented in Figure 2. The bandgap in LSO and LGSO was estimated to be about 6.5 to 6.7 eV [9]. Therefore, the transmission cutoff at approximately 6.3 eV corresponds to the electronic transitions at the fundamental absorption edge. The LGSO crystal spectrum also contains a series of narrow absorption peaks within the 4 to 5 eV range corresponding to the ^8^*S*_7/2_→^6^*P*_*J*_ transitions in Gd ^3+^. In addition, in Ce-doped crystals, the several wide absorption bands in the UV region are attributed to the 4f to 5d electronic transitions of the Ce ^3+^ ions. In LGSO:Ce, the Ce ^3+^ absorption bands are overlapped due to the higher concentration of Ce ^3+^. The weak absorption bands at 4.5 and 5.1 eV corresponding to the traces of Ce ^3+^ are observed also in nominally pure LSO and LGSO crystals.

The significant decrease of the Ce-doped crystal transmittance at about 3.2 eV could be explained by the Rayleigh scattering at Gd ^3+^- or Lu ^3+^-enriched nano- or microsize spatial inhomogeneities in crystals.

### The experimental technique

In order to characterize the studied orthosilicates, we used the technique based on self-action of the pulsed laser radiation [10] that provides an information dealing with photoinduced refractive index and optical absorption coefficient variations at the laser excitation wavelength.

**Figure 3:**
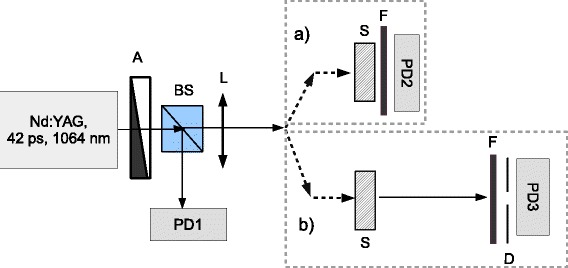
**The experimental setup for self-action technique.** **(a)** The photoinduced total transmittance variation studies due to the NLO absorption (the sample is positioned at the aperture of the photodiode in order to prevent scattering losses); **(b)** the spatial profile analysis (the beam passes the finite on-axis diaphragm in the far field) for the NLO refraction measurements.

The experimental setup is shown in the Figure 3. The excitation was performed with the fundamental harmonic of mode-locked Nd:YAG picosecond laser (42 ps FWHM, repetition rate 10 Hz) at 1,064 nm (1.17 eV) based on Sagnac interferometer that provides Gaussian spatial beam profile [11]. The peak laser intensity of incident light was varied with the gradient neutral attenuator (A). The photodiode PD1 provides an acquisition of the input laser pulse energy, the BS is a beam splitter. The focusing lens L with focal length 11 cm was used to focus the laser irradiation on the sample S. The typical aperture of the laser beam at the sample plane during the experiments was about 0.5 mm. The experimental setup allows us to measure both the total (Figure 3a) and the on-axis (Figure 3b) transmittance variations versus the incident peak laser intensity *I*_0_. In case (a), the sample S is positioned directly at the face of the wide aperture (1 cm) photodiode PD2. The PD2 signal corrected to the apparatus function provides the photoinduced variations of the total transmittance *T*(*I*_0_). The *T*(*I*_0_) can be described by the following expression considering the spatial and temporal averaging of the transmitted pulse [12]:

(1) $$ T_{t}(I_{0}) = T_{0} \frac{\ln\left(1+\Delta\alpha L_{eff}\right)}{\Delta\alpha L_{eff}}\left[\frac{1+0.228\Delta\alpha L_{eff}}{1+0.136\Delta\alpha L_{eff}}\right],  $$

where *T*_0_ is the linear transmittance coefficient at 1.17 eV (1,064 nm) as shown on the spectra (see Figure 2), *Δα*=*βI*_0_ – the photoinduced absorption variations, *β*∼*Im*(*χ*^(3)^)– effective nonlinear absorption coefficient, *χ*^(3)^ – the cubic NLO susceptibility, *α* - the linear absorption coefficient, *L*_*eff*_=(1− exp(−*αL*))/*α* is a self-action effective length [12], and *L* - the sample thickness. The expression (1) is valid for the thin layers without beam spatial profile redistribution that was checked for our crystals.

We have introduced the IR-passing filter F at the PD2 aperture to cut off a weak visible range glow that was observed by naked eye during the pulsed IR high level excitation. The observed effect needs additional experimental study and it is out scope of the present work.

**Figure 4:**
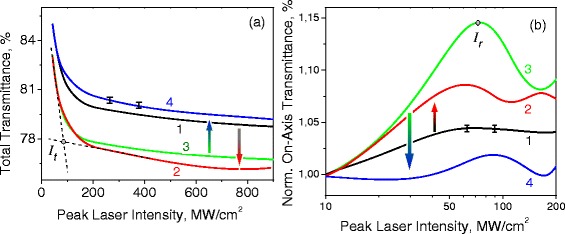
**The photoinduced variations of the total and the on-axis transmittances versus the peak laser intensity.** The photoinduced variations of the total **(a)** and the on-axis **(b)** transmittances versus the peak laser intensity at 1,064 nm for the orthosilicate single crystals: 1 - LSO, 2 - LSO:Ce, 3 - LGSO, 4 - LGSO:Ce. The arrows demonstrate the impact of Ce ions incorporation on the absorptive and refractive NLO response of the LSO and LGSO crystals. The magnitudes of the peak laser intensities *I*_*t*_ and *I*_*r*_ correspond to the experimental dependencies slope changes.

For the setup (b) case, the PD2 signal allows us to interpret the on-axis transmittance in the far field *T*_*a*_(*I*_0_). The *T*_*a*_(*I*_0_) provides an information about the convergence/divergence of the laser beam in the far field that is caused by the lensing effects (self-focusing/defocusing) in the media [10,13] and can be described in terms of the peak photoinduced phase shift after the sample *Δϕ*(*I*_0_). According to the model for the cubic refractive NLO response with negligible photoinduced absorption [13], the *Δϕ*(*I*_0_) is proportional to the photoinduced refractive index *Δϕ*(*I*_0_)=*kL*_*eff*_*Δn*(*I*_0_)∼*Re*(*χ*^(3)^))*I*_0_ (*k* - the wave vector). On the base of the Gaussian decomposition approach and consequent spatial and temporal averaging of the transmitted pulse, the on-axis transmittance *T*_*a*_(*I*_0_) in the far field can be presented as an expansion into *Δϕ*:

(2) $$ T_{a}(I_{0}) = S \left(1+C_{1}\Delta\phi\left(I_{0}\right)+C_{2}\left(\Delta\phi\left(I_{0}\right)\right)^{2}+\dots\right),  $$

where coefficients *C*_*n*_ are determined by the geometry of the experiment:

(3) $$  \begin{aligned} C_{1}&=\frac{1}{S\sqrt{2}}\exp\left[\frac{-4{r_{0}^{2}}\left(3+b^{2}\right)}{a^{2}\left(9+b^{2}\right)} \right]\sin\left[\frac{8b{r_{0}^{2}}}{a^{2}\left(9+b^{2}\right)}\right] \\ C_{2}&=\frac{1}{3S\sqrt{3}}\left(\exp\left[ \frac{-6{r_{0}^{2}}\left(5+b^{2}\right)}{a^{2}\left(25+b^{2}\right)} \right] \cos\left[ \frac{24b{r_{0}^{2}}}{a^{2}\left(25+b^{2}\right)}\right] \right.\\&\left.\quad- \exp\left[ \frac{-6{r_{0}^{2}}\left(1+b^{2}\right)}{a^{2}\left(9+b^{2}\right)}\right] \right), \end{aligned}  $$

where *S* - is the aperture linear transmittance, *r*_0_ - the diaphragm radius, *a* - the beam radius at the sample plane, and *b* - the ratio of the geometric focusing to the diffraction broadening (see [7]). In order to compensate the impact of the photoinduced transmittance variation on refractive NLO response, we normalize the on-axis transmittance in the far field on total transmittance of the sample. The verification of the procedure was given in [13]. Fitting of the experimental data of the total/on-axis transmittances versus the peak laser intensities (Figure 4) within formulae (1) and (2) correspondingly provides magnitudes of *Δα* and *Δn* as well as Im(*χ*^(3)^) and Re(*χ*^(3)^) for the different excitation ranges.

## Results and discussion

### The experimental results

The photoinduced total and on-axis transmittance variations versus the peak laser pulse intensity are presented in Figure 4a,b correspondingly for the studied samples. Each experimental curve corresponds to transmittance data acquisition for about 5,000 laser shots with varied peak intensities governed by the attenuator. We have shown typical error bars at different excitation levels which relative magnitude does not exceed 0.2% for the total transmittance and 0.5% for the on-axis transmittance. The schematic arrows demonstrate the impact of Ce ions incorporation on the absorptive and refractive NLO responses for each of single crystalline hosts - LSO and LGSO.

Analysis of the absorptive NLO response (see Figure 4a) has shown the photodarkening effect exhibition for the all samples with two characteristic slopes: the most efficient in the initial excitation range *I*<*I*_*t*_ that essentially reduces at higher intensities *I*>*I*_*t*_. For the LSO:Ce data, we have shown the characteristic tangents (dashed lines). The intensity magnitude *I*_*t*_ for the tangent intersection point can be treated as a switching threshold from the initial effective response to the more saturated one. The fitting results for the Im(*χ*^(3)^) according model function (1) in different excitation levels *I*_0_≤*I*_*t*_ and *I*_0_>*I*_*t*_ both with the threshold intensity *I*_*t*_ are presented in Table 1. Substitution of Lu with Gd atoms do not significantly affect the *I*_*t*_ magnitude (*I*_*t*_[*LSO*]∼*I*_*t*_[*LGSO*]). The incorporation of Ce ions leads to the about 6% *I*_*t*_ reduction for the LSO host and to an approximately 10% rise for the LGSO one. Both nominally pure hosts have the similar NLO response in the intensity range *I*_0_≤*I*_*t*_*Im*(*χ*^(3)^)∼4.2·10^−12^ esu. It reduces an order of magnitude (∼4.1·10^−13^ esu) after the initial effective photodarkening response saturation at *I*_0_>*I*_*t*_.

**Table 1:** **The imaginary and real parts of the cubic NLO susceptibility**

**Ranges**	***I***_*t*_ **, MW/cm** ^**2**^	***I***_0_ **≤** ***I***_*t*_	***I***_0_ **>** ***I***_*t*_	***I***_*r*_ **, MW/cm** ^**2**^	***I***_0_ **<** ***I***_*r*_	***I***_0_ **>** ***I***_*r*_	***FOM***
**Sample**		***I*** ***m*** **(** ***χ*** ^**(3)**^ **),10** ^**−12**^ ** esu**			***R*** ***e*** **(** ***χ*** ^**(3)**^ **),10** ^**−10**^ ** esu**		
LSO	92.9	4.2	0.41	68.4	0.9	−0.1	0.6
LSO:Ce	87.6	5.3	0.43	61.6	1.8	−1.0	1.4
LGSO	91.7	4.2	0.41	73.8	2.9	−2.0	1.1
LGSO:Ce	100.6	3.6	0.42	88.2	0.4	−0.6	0.3

The imaginary and real parts of the cubic NLO susceptibility *χ* ^(3)^ estimated for different intensity ranges, the peak laser intensities magnitudes of the absorptive *I*_*t*_ and refractive *I*_*r*_ responses slope changes, figure of merit (*FOM*) for the *I*_*0*_ <*I*_*r*_ range.

The incorporation of Ce ions induces the opposite trend in the absorptive NLO response at *I*_0_≤*I*_*t*_: an enhancement of the photoinduced absorption variations efficiency by about 30% for the LSO crystalline matrix, and its reduction by up to 20% for the LGSO one. At higher excitation levels *I*_0_>*I*_*t*_, the efficiencies of photodarkening effect for the Ce doped crystals are similar and exceed the corresponding one for the nominally pure hosts by up to 5%.

The refractive NLO response (see Figure 4b) has a definite distinction for the LGSO:Ce sample in comparison with the rest ones. It demonstrates weak self-defocusing effect *Δn*<0 (*Re*(*χ*^(3)^)∼−1.1·10^−11^ esu) that corresponds to slight on-axis transmittance reduction in the far field up to intensities of 25 MW/cm ^2^. At higher intensities, the LGSO:Ce response becomes similar to other samples that demonstrate self-focusing effect *Δn*>0 (*Re*(*χ*^(3)^)∼10^−10^ esu): rise of the on-axis transmittance in the far field versus the peak intensities due to the photoinduced lensing effect in the crystals. We present the plot in semilogarithmic scale in order to resolute the manifestation of the initial self-defocusing effect in the LGSO:Ce.

The experimental on-axis dependencies have a local maximum at peak intensity *I*_*r*_; after that, the slopes turn to the opposite sign. The fitting results for the refractive NLO response efficiency *Re*(*χ*^(3)^) according to the model (2) at different excitation levels *I*_0_<*I*_*r*_ and *I*_0_>*I*_*r*_ both with the intensity *I*_*r*_ are presented in Table 1. In general the *I*_*r*_<*I*_*t*_ for the studied samples: *I*_*r*_ ∼ 0.7/0.8 *I*_*t*_ for the LSO/LGSO-based crystals.

One can see that the efficiency of the self-focusing effect at *I*_0_<*I*_*r*_ drastically depends both on Gd/Lu atoms substitution and on Ce ions incorporation. At higher intensities *I*_0_>*I*_*r*_, it turns to self-defocusing phenomenon with the reduced efficiency that differs for the all studied crystals. The Gd/Lu atoms substitution provides the approximately three times enhancement of *Re*(*χ*^(3)^[*LGSO*])/*Re*(*χ*^(3)^[*LSO*]) refractive NLO response for *I*<*I*_*r*_ range, and more than an order of magnitude difference in self-defocusing efficiency |*Re*(*χ*^(3)^)| effect (*I*>*I*_*r*_). It is worth to note that a corresponding rise of the *I*_*r*_ is about 8% only.

The Ce ion incorporation causes the similar trends for refractive NLO response in comparison with the absorptive one, but with much pronounced efficiency of the effect manifestation. In the LSO crystals case, the Ce ions admixture leads to the two times enhancement for the self-focusing efficiency (*Re*(*χ*^(3)^[*LSO*:*Ce*])/*Re*(*χ*^(3)^[*LSO*])∼2) at *I*_0_<*I*_*r*_ and up to 10 times for the self-defocusing one at *I*_0_>*I*_*r*_. Unlike, for the LGSO crystal the *Re*(*χ*^(3)^) magnitudes reduction was observed: *Re*(*χ*^(3)^[*LGSO*:*Ce*])/*Re*(*χ*^(3)^[*LGSO*])∼0.15 at *I*_0_<*I*_*r*_, and |*Re*(*χ*^(3)^[*LGSO*:*Ce*])/*Re*(*χ*^(3)^[*LGSO*])|∼0.3 at *I*_0_<*I*_*r*_. Thus, basing on the NLO absorption efficiency variation range of 20% to 30% due to Lu/Gd substitution and Ce ion incorporation impact, the refractive NLO response differs by more than one order of magnitude at the crystal host composition modification.

We have checked the reversibility of the absorptive and refractive NLO response in a two-stage experiment: (*i*) for the first one, we increase the laser intensity from the minimum to the maximum magnitude, and (*ii*) afterwards, we reduce the intensity in the opposite way for the same irradiation spot at the sample. The obtained reversibility of the NLO total and on-axis transmittance variations means the absence of both the photoinduced refractive index and optical absorption modifications in the crystals and the accumulation effect manifestation.

### Discussion

Recently, it has been shown that an efficient manifestation of the NLO refractive response of the metal oxide nanocomposites in the transparency range due to the resonant electron excitation from the deep traps [7,8] in the gap attributed to intrinsic oxygen vacancies [14,15]. For anatase nanocomposites, the correlation between giant NLO response efficiency and their photocatalytic activity was established [16], density of the radicals on the developed surface [17], and their sensory properties [18]. The proposed approach have been successfully applied for the prediction, elaboration, and realization of the nanocomposites with improved frequency conversion properties based on KH _2_*PO*_4_ (KDP) single crystals with incorporated TiO _2_ nanoparticles [19,20]. Furthermore, the strong correlation of the defect concentration studied by neutron diffraction with the photoinduced refractive index and optical absorption coefficient variations were observed in bulk ZnO crystals [21] due to the CW laser resonant excitation of the carriers from the valence band into the deep trap states.

As specified in the other metal oxides, the NLO response of the studied orthosilicates is mostly determined by the traps’ activation within the laser pulses at 1,064 nm. Thermoluminescence study of LSO:Ce crystal have shown that the observed traps had depth (the energy difference between the conduction band edge and the energy level of the trap) in 1 to 1.7 eV range which can be attributed to the oxygen vacancies [22]. As it was recently shown by the authors [6,23], the trap depth related to the oxygen vacancies is about 1 eV in LSO scintillator.

The defects in rare earth orthosilicates are formed during crystal growth from melt in atmosphere of inert gas (Ar or He) at 2,000 °C temperature. It is impossible to avoid completely a deficiency by oxygen in melt and crystal in such conditions. Introduction of even 1 to 2 vol.% of oxygen into growth atmosphere results in Ir crucibles oxidation and destruction. Regarding the concentration of filled traps in LSO:Ce, it was evaluated as approximately 10 ^12^ mg ^−1^ [23]. Amount of filled oxygen vacancies in LGSO:Ce have not been evaluated though it is surely smaller, because thermoluminescence intensity falls with Gd addition [3]. The incorporation of cerium to the LSO/LGSO host results in formation of additional recombination center Ce ^3+^. The detrapping-recombination process, in this case, can be described as follows [6]: (*i*) the promotion of the trapped electron activated by the picosecond laser pulse at 1,064 nm (1.17 eV) to the CB; (*ii*) detrapping of the electron by Ce ^3+^ 5d _1_ state; (*iii*) transition of the electron to Ce ^3+^ 4f ground state with visible light emission (the glow was observed during the experiment).

It is worth to note that for most oxides, it needs to transfer at about 50 eV displacement energy for the permanent color F ^+^ center creation [24]. In our case of the near IR range excitation, the laser radiation creates transient defects with high polarizability probably due to the perturbation of polaronic states. To our point of view, within picosecond range laser pulses, we provide an efficient readout of the trapped electron response. The phenomenon is similar to the one observed in TiO _2_ doped KDP crystals [10].

It should be stated that the probable electron excitation and transfer from the valence band to the empty levels of the impurity or other defects can be neglected in our case. During the experiments, we have observed blue-green luminescence around the laser beam. This obviously corresponds to Ce ^3+^ luminescence. The bandgap in this material is approximately 6.5 eV, and the excited levels of Ce ^3+^ are situated by 0.5 eV lower than conductance band edge. Laser beam energy is not enough to excite electrons from energy levels near the top of valence band to Ce ^3+^ excited levels.

The cerium incorporation also leads to the considerable lattice relaxation of the neighbor oxygen ions nearby the Ce ^3+^ ions due to the larger ionic radius of Ce ^3+^ versus Lu ^3+^ [6,23] that significantly affects the detrapping-recombination process. In the case of the LSO crystal, it increases the NLO response efficiency unlike the LGSO one.

One should note the efficient enhancement of the refractive NLO response observed at Lu/Gd substitution. It can be attributed to the mentioned nano- or microsize spatial Gd ^3+^ inhomogeneities in the LGSO crystals. The increased amount of the oxygen vacancies at their interface leads to the higher specific polarizability of the inhomogeneities that results in significant refractive NLO response enhancement.

In order to estimate the NLO refractive index efficiency, we use the dimensionless parameter - the figure of merit *FOM*=|*Δn*/(*Δαλ*)| [8]. It reflects the photoinduced refractive index gain toward the photoinduced absorption variation at a corresponding wavelength. The estimated *FOM* magnitudes for the initial laser peak intensity range *I*_0_<*I*_*r*_ are presented in Table 1. The Ce ion incorporation leads to the rise of the refractive contribution manifestation for the LSO crystal unlike the LGSO one. The obtained *FOM*∼1 magnitudes indicate that the NLO refractive and absorptive responses have the same origin due to the resonant oxygen vacancy excitation. The fact that typical saturation intensity for the primary NLO refractive response is less than the saturation intensity of the photoinduced absorption *I*_*r*_<*I*_*t*_ also confirmed our suggestion.

We suggest to apply the proposed nonlinear optical diagnostics approach based on the oxygen vacancy excitation within the picosecond laser pulses at 1,064 nm (1.17 eV) and NLO refractive index response readout. This approach is promising for the composition modification studies in orthosilicate-based scintillators, as well as in persistent phosphors due to the similar position of the traps at about 1 eV below the CB [25-27].

## Conclusions

The nonlinear optical response characterization of the LSO and mixed LGSO (nominally pure and doped with Ce ions) single crystals were performed under the picosecond laser excitation at 1,064 nm. The photoinduced absorption effect with efficient self-focusing of the laser beam with relevant refractive NLO response at about *Re*(*χ*^(3)^)∼10^−10^ esu was observed. The magnitude of the response efficiency is sensitive to the substitution of the Lu atoms by Gd, as well as to the admixture of Ce in the LSO/LGSO crystalline host. While in the case of the photoinduced absorptive variations, it is within the limits of about 20% to 30%, the photoinduced refractive response showed much higher sensitivity to the crystal composition due to the *Re*(*χ*^(3)^) rise/reduction by about 3 to 10 times. Both the effects are based on resonant electron excitation from the deep traps in the gap attributed to intrinsic oxygen vacancies. We suggest to apply the mentioned NLO diagnostics based on picosecond range pulsed laser radiation self-action technique at 1,064 nm (1.17 eV) for the orthosilicate single crystals characterization to improve their optical quality and scintillation efficiency.
